# Influence of clone and nitrogen application level on quality of green tea in some selected tea (*Camellia sinensis* (L.)O. Kuntze) in Southwest Ethiopia

**DOI:** 10.1016/j.heliyon.2022.e10179

**Published:** 2022-08-14

**Authors:** Tesfaye Benti, Adugna Debela, Yetenayet Bekele, Sultan Suleman

**Affiliations:** a Department of Horticulture College of Agriculture in Mizan Tepi University, Southwest, Ethiopia; b Ethiopia Coffee and Tea Authority, Oromia, Ethiopia; c Department of Postharvest Management of Jimma University College of Agriculture, Oromia, Ethiopia; d School of Pharmacy, Jimma University Director, Jimma University Laboratory of Drug Quality, Oromia, Ethiopia

**Keywords:** Antioxidant, Caffeine, Camellia sinensis, Cup quality, Green tea, Polyphenol

## Abstract

The biochemical constituents and organoleptic characteristics of *Camellia sinensis* (L.) O. Kuntze clones are not studied well in Ethiopia. The study aims to evaluate the polyphenols, caffeine, antioxidant content, and cup quality of clones at different nitrogen application rates and identify suitable clones and processes for daily consumable antioxidant-rich green tea. The experiment consisted of five clones (6/8, BB-35, 11/56, 11/4, and 12/38) and five nitrogen application rates (i.e., 0, 75, 150, 225, and 300 kg ha^−1^) under a split-plot design and was replicated three times. The biochemical constituents and overall quality of green tea were examined using HPLC, chemical analysis, and a cup taster. The results indicate that the polyphenol content increased slightly (P < 0.018) as the nitrogen application rate increased, with a weak correlation (r = 0.387). The caffeine content varies from 1.82 to 3.06%. Clone BB-35 scored the highest (3.06%), and clone 6/8 scored the lowest caffeine content in all nitrogen treatments. The total nitrogen content varied between 2.27 and 4.01 mg g^−1^ and slightly increased as the nitrogen application rate increased (r^2^ = 0.798). The antioxidant activity showed a significant variation (P < 0.001) among clones, and the tested clones had a high antioxidant inhibition percent ranging from 51.9 to 66.5%. The cup quality, aroma, liquor color, and dry leaf appearances of green tea were positively correlated (r = 0.68, r = 0.70, r = 0.48, and r = 0.30), and the overall quality of green tea improved as the nitrogen application rate increased. Clones 6/8 and 11/4 recorded the highest overall quality above the total mean value, whereas clones BB-35, 12/38, and 11/56 scored below the total mean value (77.68%). Clone 6/8 produced an excellent full aroma, balanced bitterness, and a slightly sweeter cup in all nitrogen treatments than other clones. Moreover, this clone contains less caffeine and high polyphenol content in high nitrogen treatment. The second-best, clone 11/4, has a good marketable green liquor, aroma, and dry-made tea appearance, but it is slightly bitterer than clone 6/8. The research concluded that clone 6/8 can produce a more acceptable and high-quality green tea at 300 kg N ha^−1^.

## 1 Introduction

The tea plant (*Camellia sinensis* (L.) O. Kuntze) is the most popular, non-alcoholic, and caffeine-containing beverage crop in the world. The plant originated in Southeast Asia around the intersection of latitude 29^0^N, and longitude 98^0^E, at the point of confluence of the lands of Northeast India, North Burma, Southwest China, and Tibet [1].

Ethiopian tea plantations are concentrated in the Southwest highlands, and the cultivation began in 1927 by the Canadian Catholic missionary Father George Holland. He brought tea seeds from Kenya and planted them around Bonga. The second tea seed was imported in 1928 from India by the British general councilor stationed at Gore and planted in the Gumaro tea plantation near Illubabor [2, 3]. However, the first commercial tea plantation was established in 1957 at 25ha, expanded to 3175.31ha in 2020, and produced 7767.6 tonnes y^−1^ on average, with 86 percent consumed locally and the remaining 14 percent exported over the last ten years. Even so, the quality, quantity, and type of tea are insufficient to meet the ever-growing demand of the local market*.* For instance, the production volume of Ethiopian black tea (8543.25 tonnes) was too small to compare with Kenyan (473,300 tonnes) in 2018, which differs by a ratio of 1:55 tonnes.

The tea plant is widely adaptable to geographical areas with high variation in climate and physical features, which affect the growth rates, yields, and quality [4, 5]. However, the quality and composition of made tea vary with the season, climate, agricultural practices, varieties of plants, age and types of leaf, processing methods, and geographical location of the tea plant [6, 7, 8, 9]. Nitrogen is the most deficient mineral element in the soil [10] and it is the main factor in achieving good quality and high yields in the production of green tea [11, 12]. Hence, the continuous plucking and pruning removed a huge amount of nutrients from the tea field [13]. To overcome the problem, East African tea producers used 100 to 250 kgNha^−1^y^−1^ fertilizer as NPKS 25: 5: 5: 5 or NPK 20: 10: 10 [14,15]. However, the tea plant responded to 500 kg of nitrogen per hectare, and its economic returns peaked at 200–300 kgNha^−1^ [16]. Ethiopia's tea industry used 150 kgNha^−1^y^−1^ in the ratio of NPK as 20:10:10 for the production of black tea, below the economic application range of East Africa and a reason for producing uneven green leaves, which is a poor material for the production of green tea.

Camellia sinensis var. sinensis is used for green tea production, whereas black tea is produced from the assamica variety [10, 17, 18]. Camellia sinensis var. sinensis contains low polyphenols and gives the required astringency to the infusion without being excessively bitter [17, 19], and this clone is only used for academic and research purposes in Ethiopia due to its poor growth and yield performance. The other variety (Camellia sinensis var. assamica) is commercially grown in different proportions, i.e., mixed clones cover around 38% of the total cultivated (3175.31ha) land, followed by clones like 11/56 (19%), BB-35 (14%), 11/4 (10%), 6/8 (10%), and 12/38 (3%), and the remaining 6% covered by clones like SR, 18/49, GUM, FNF, S15/10, 31/11, 31/12, 31/8, and TN14/3. These clones are only used to make black tea, whereas green tea production is still in trial, and the product has an undesirable bitterness, which is not preferred by children and young men. Hence, more than 110 million Ethiopians are not familiar with the benefits of green tea intake.

Green tea is rich in antioxidants and it plays a role in the destruction of free radicals and reactive oxygen species in human bodies [20]. Some cohort studies in far-east countries like China, Vietnam, South Korea, and Japan showed that green tea reduces the occurrence and risk of a variety of disorders, such as breast cancer, prostate cancer, lung cancer, pancreatic cancer, colon cancer, esophageal cancer, skin cancer, coronary heart disease, and weight loss [21]. Green tea is the highest and cheapest daily antioxidant given as a mass treatment for the population to remove free radicals, which cause aging and death of the cell and unwanted cell division [22]. The rapid development of different types of cancer epidemics has been observed, especially breast and cervical cancer being the most common substantial public health threats in Ethiopia [23, 24]. Such new cancer prevalence rates increase with population growth and a change in social feeding habits. Therefore, this study intends to identify suitable clones and process antioxidant-rich green tea in Ethiopia.

## 2 Materials and methods

### 2.1 The study area

The study was conducted at the Wushwush tea plantation, located 472 km from Addis Abeba (in Ethiopia's Southwest highlands; 1720 to 1990 masl; 7° 18′N; 36°E°). The areas received annual rainfall (1640–2000mm) during the eight wet months (March–November) and four dry months (December-mid-March), with an average temperature of 25 °C (max.) and 12 °C (min.).

### 2.2 Experimental design

The experiment was carried out in a split-plot arrangement by placing clones in main plots and applying nitrogen application rates in sub-plots; the main plot contained five clones (6/8, BB-35, 11/56, 11/4, and 12/38), and the subplot had five nitrogen treatments (0, 75, 150, 225, and 300 kg N ha^−1^). The 28-year-old tea bushes were chosen and pruned one year before the experiment, and all phosphate and potassium were applied at a rate of 150 kg ha^−1^ as NPK 20:10:10, and each plot has a total of four rows with a size of 6.0 × 3.6 m on an established tea farm with a spacing of 1.2 × 0.6 m. Early in the morning, a bud +1 and a bud +2 shoot were harvested and steamed at 100 °C for less than 120 s to arrest fermentation, and the steamed leaves were spread in a thin layer on a withering trough, exposed to cool air for 6–8 h to reduce moisture and the grassy nature of the leaves, and the withered leaves were cut, tear, and crushed using a small CTC and drier with an inlet temperature of 70–90 °C for 20 s. Then, the green-made tea samples were sorted and packed for the next step of chemical analysis.

### 2.3 Method of data collection

#### 2.3.1 Cup taste

The ground (3g) tea samples were infused with 150 ml of boiling water for 5 min, and the tea liquor was poured into 200 ml tea bowls. The samples were served to five panelists, and the samples were leveled randomly as clones (A, B, C, D, and E), and the panelist applied cold water to rinse their mouths before and after each cup of tea and asked them to rate all green tea quality parameters based on a maximum score of 100, of which 10% was used for the dry tea appearance, 10% for the aroma, 30% for the liquor color, 30% for the taste, and 20% for the infused leaf. 200g of dry tea samples were placed on white paper and rated out of 10%. Finally, the individual cup taster score result was taken independently, and the mean value of the five scores was taken as the score of a sample.

#### 2.3.2 Extraction and determination of total polyphenol

The polyphenol sample was extracted according to ISO 14502-1-2005, as described by [25]. In brief, the ground tea sample (0.200 + 0.001g) was weighed in an extraction tube, and test tubes containing 5ml of 70% methanol solution were kept in a 70 °C water bath. The extract was mixed and heated at 70 °C in a vortex for 10 min. The extract was allowed to cool at room temperature before being centrifuged at 3500 rpm for 10 min to separate the supernatant, which was decanted in a graduated tube. The extraction step was repeated twice. Both the extracted pool and the volume were adjusted to 10 ml with cold 70% methanol. Then, 1 ml of the extract was diluted to 100 ml of water.

According to ISO 14502-1-2005, the polyphenol content can be determined using the Folin-Ciocalteau phenol reagent and gallic acid, as described by [25, 26]. In brief, 1.0 ml of the diluted sample extract was transferred into duplicate tubes containing 5 ml of a 1/10 dilution of Folin-Ciocalteau reagent in water, then 4 ml of a sodium carbonate solution of 7.5% w/v (37.5 g diluted with 500 ml of distilled water) was added, and the tubes were allowed to stand at room temperature for 60 min before a blue color absorbance was measured at 765nm. The total polyphenol concentration was expressed as gallic acid equivalents in g/100g of material.

#### 2.3.3 Extraction and determination of antioxidant capacity

The ground (2g) tea samples were infused with 200ml of distilled boiling water for 5 min at a temperature of 80 °C, as described in [27], Then the sample was filtered through a nylon mesh followed by a filter paper (Whatman No.42), and the extracts were kept at a cool temperature for 1 or 2 h until the next step of the analysis. The tea extract (100μl) was added to 2.9 ml of DPPH (2,2-diphenyl-1-picrylhydrazyl radical) solution (2μg DDPH diluted to 100ml with 80% methanol). The solution was mixed with a vortex and incubated for 30 min in darkness at room temperature, and the absorbance was measured at a wavelength of 517nm, using methanol as a reference. The values of percent inhibition were calculated using the following formula [28].

% Inhibition = [(A_0_ – A_t_) / A_0_] × 100

Where: - **A**_**0**_ = absorbance of blank (control) sample (t = 0 min)

**A**_**t**_ = absorbance of extract sample (t = 30 min)

The DPPH mediated percent inhibition method calculates the concentration difference between the absorbance of a blank sample and the absorbance of an extracted sample. The changes in color (from deep-violet to light-yellow) were measured at 515 nm using a UV/visible light spectrophotometer. DPPH has been extensively used as a free radical to evaluate reducing substances [29]. It is a useful reagent for investigating the free radical-scavenging activities of polyphenol compounds [30].

#### 2.3.4 Determination of caffeine in tea plant

Caffeine analyses were done at Jimma University Quality Drug Control Laboratory with the following protocol: The ground (125g) tea sample was extracted with 100 ml of acetonitrile-water (1:1 v/v), then the flask was immersed in an ultrasonic bath (Thornton model T50, Brazil) at room temperature for 10 min, and the mixture was centrifuged at 3500 rpm for 10 min and passed through a funnel lined with Whitman 42 before injection into Agilent 1260 series HPLC Agilent Technology, using a Hc = -009 (4.6mm x 15-cm x 5μm) reversed-phase (RP) for analysis. The mobile phase A consisted of phosphoric acid 85% (v/v), acetonitrile 15% (v/v), and mobile phase B consisted of water 85% (v/v), acetonitrile 15% (v/v). The flow rate is controlled at 1.5 ml/min with an injection volume of 20 ml. The column operated at 40 °C, and UV spectra peaks were detected at 273 nm. The chromatographic peaks in the samples are identified by comparing their retention times with the chemical standards used. The caffeine level was determined using a solvent gradient and Shimadzu HPLC with a UV detector as described in the International Organization for Standardization [31].

#### 2.3.5 Determination of leaf nitrogen content

Fifty mature leaves were taken from each plot and oven-dried at 65 °C for 48 h. Dry leaf samples were taken from the oven after the leaves were well dried and immediately grounded into a leaf powder, then packed in an airtight polyethylene bag before nitrogen analysis. The total nitrogen concentration is determined by the micro-Kjeldahl method.

## 3 Result and discussion

### 3.1 Polyphenol analysis

The polyphenol content of the clone was significantly affected by the type of clone and different nitrogen treatments (P < 0.018) and slightly increased as the nitrogen application rate increased. Clones 6/8, 11/4, and BB-35 scored the highest (26, 24.07, and 23.8%) polyphenol content at 300 kg N ha^-1^, while clones 11/56 and 12/38 scored the lowest (22.6 and 22.8%) polyphenol content at 225 kg N ha^-1^, respectively (Table 1).

**Table 1:** Variation of clones in polyphenol content at different nitrogen rates.

Items	Clone	Nitrogen (kg ha^−1^)					
		0	75	150	225	300	Mean
Polyphenols content (g/100 g)	BB-35	23.1^defg^	23.5^cde^	23.2^defg^	22.3^hijkl^	23.8^bcd^	23.19^b^
	6/8	22.5^ghij^	22.3^hijk^	24.07^bc^	24.5^b^	26.0^a^	23.87^a^
	11/4	22.7^ghi^	22.7^fghi^	23.6^cd^	23.4^cdef^	24.07^bc^	23.3^b^
	11/56	22.3^hijk^	21.7^kl^	21.9^ijk^	22.6^ghij^	21.97^jkl^	22.09^c^
	12/38	20.7^m^	21.5^l^	21.5^l^	22.8^efgh^	22.3^hijkl^	21.76^c^
	Mean	22.25^c^	22.35^c^	22.86 ^bc^	23.11 ^ab^	23.63^a^	22.85
	C.V. (%)	3.89	LSD (≤ 0.05) 0.753				

Different letters in the same columns indicate the difference between the two mean statistically significant (P < 0.01) for each of the treatment combinations.

The clones in southwest Ethiopia showed similar significant differences in total polyphenol (24.17–30.82 mg GAE/g) [32]. Also, the mean percentage comparison of Kenya clones (TRFK 6/8 and BBK 35) has the highest mean total polyphenols of 25.90 and 25.75% [33]. Hence, the polyphenol content in green leaves showed minimal variations within the same clone but significant differences among clones [34]. In general, the tested clones have similar polyphenol content, but clones 6/8, 11/4, and BB-35 scored a high polyphenol content above the total mean (22.85 %) at 150, 225, and 300 kg nitrogen applications, whereas clones 11/56 and 12/38 scored a low polyphenol content below the total mean value in all nitrogen applications. The mean comparison of polyphenol over a control treatment was increased by 0.45, 2.74, 4.13, and 6.2% as nitrogen application rates increased from 75 to 300 kg N ha^−1^ (Table 1) and was weakly correlated (r = 0.387) and a similar positive relationship between leaf polyphenol content and level of nitrogenous fertilizer application was reported by [35].

### 3.2 Caffeine analysis

The caffeine content of each clone was significantly affected by different nitrogen application rates (P < 0.0001). The caffeine contents of green tea samples range from 1.82–3.06%, with the highest 3.06% of caffeine scored by clone BB-35, followed by clones 12/38 (2.97), 11/56 (2.66), 11/4 (2.57), and 6/8 (2.35%), produced at 300 kg N ha^−1^ (Table 2). The lowest caffeine content was scored by clone 6/8 in all nitrogen treatments. It implies that clonal variation has an impact on caffeine content.

**Table 2:** Variation of clones in caffeine content at different nitrogen rates.

Items	Clone	Nitrogen (kg ha^−1^)					
		0	75	150	225	300	Mean
Caffeine (%) content	BB-35	2.32^fghi^	2.44^efghi^	2.77^abcde^	2.94^abc^	3.06^a^	2.71^a^
	6/8	1.82^k^	1.94^jk^	1.92^jk^	2.13^hijk^	2.35^fghi^	2.03^c^
	11/4	2.21^ghij^	2.09^ijk^	2..28^ghij^	2.45^defghi^	2.57^cdefg^	2.32^b^
	11/56	2.15^hijk^	2.1^ijk^	2.23^ghij^	2.53^defg^	2.66 ^bcdef^	2.33^b^
	12/38	2.49^defgh^	2.43^efghi^	2.57^cdefg^	2.8^abcd^	2.97^ab^	2.65^a^
	Mean	2.197^d^	2.2^d^	2.35^c^	2.57^b^	2.72^a^	2.41
	C.V. (%)	4.83	LSD (≤0.05) 0.375				

Different letters in the same columns indicate the difference between the two mean statistically significant (P < 0.0001) for each of the treatment combinations.

The variations in the caffeine content could be the effect of the genetic diversity of the clones [36]. Also, the mean comparison of caffeine contents was increased (0.14, 6.96, 16.98, and 23.81%) as nitrogen application increased from 75 to 300 kg N ha^−1^ (Table 2), and is positively correlated (r = 0.579). Here, the caffeine content of the green tea sample significantly increases as the nitrogen application rate increases. Caffeine levels increased in all locations with an increase in nitrogen rate [37], with a high rate of nitrogenous fertilizers [38], and with a linear increase in tea shoots with increased nitrogen fertilization [39].

**Figure 1:**
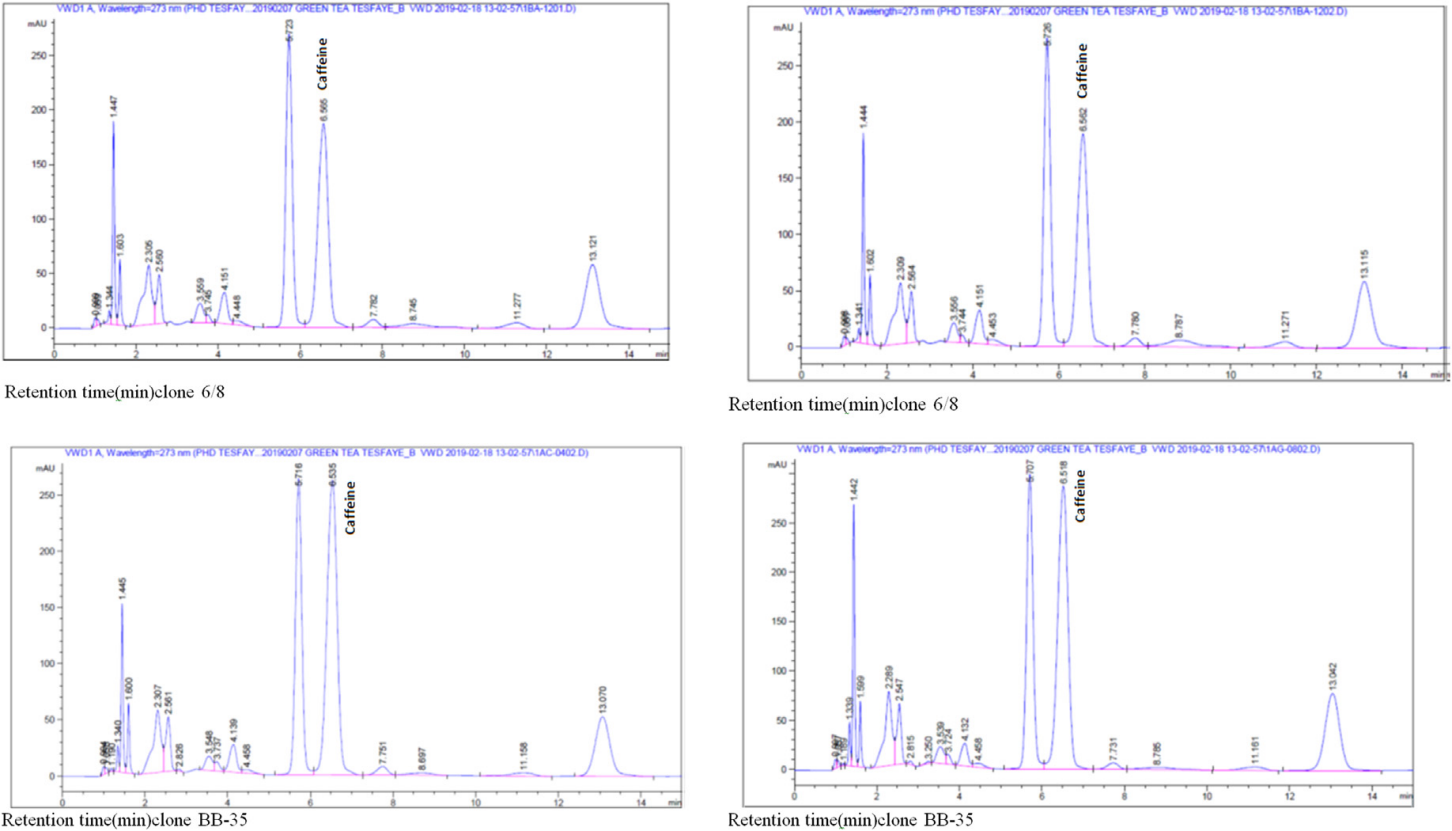
A sample HPLC profiles of caffeine retention time at 273nm for clones 6/8 and BB-35.

The retention time of green tea showed a clear difference among the clones (Figure 1). Clone 6/8 scored the lowest caffeine, and clone BB-35 scored the highest caffeine content compared to other clones. The caffeine content of clones ranged from 1.82 to 3.06%, and this value agreed with the literature quoted; tea leaves contain about 2–4% caffeine (% in dry weight) [40].

### 3.3 Leaf nitrogen content

The leaf nitrogen content of the clone was significantly affected by the nitrogen application rates at (P < 0.001) and slightly increased at (r^2^ = 0.798). The total nitrogen content of the leaf sample varied from 2.27 to 4.01 mg g^−1^. The mean comparison of leaf nitrogen content showed increments of 2.48, 2.95, 3.47, 3.87, and 4.01% as the nitrogen application rate increased (Table 3). The correlation analysis showed that leaf nitrogen content was significantly correlated with cup quality (r = 0.68), aroma (r = 0.70), infusion liquor (r = 0.53), dry leaf appearance (r = 0.30), and overall quality (r = 0.67). Also, the quality of green and black tea is influenced by the level and form of nitrogen supply to tea plants [11].

**Table 3:** Variation of clones in leaf nitrogen content at a different nitrogen rate.

Items	Clone	Nitrogen (kg ha^−1^)					
		0	75	150	225	300	Mean
Leaf nitrogen Content (%)	BB-35	2.4^klmn^	2.49^jklmn^	3.11^efgh^	3.41^cde^	3.76^abc^	3.03^b^
	6/8	2.48^lmn^	2.74^hijkl^	3.47^bcde^	3.87^ab^	4.01^a^	3.32^a^
	11/4	2.46^jklmn^	2.95^fghi^	3.28^defg^	3.59^bcd^	3.83^ab^	3.22^a^
	11/56	2.3^mn^	2.67^ijklm^	2.85^hij^	3.29^def^	3.34^de^	2.89^b^
	12/38	2.27^n^	2.39^lmn^	2.66^ijklmn^	2.79^hijk^	2.89^ghi^	2.6^c^
	Mean	2.38^e^	2.64^d^	3.07^c^	3.39^b^	3.57^a^	3.01
	C.V. (%)	6.309	LSD (≤0.05) 0.395				

Different letters in the same columns indicate the difference between the two mean statistically significant (P < 0.001) for each of the treatment combinations.

### 3.4 Antioxidant analysis

The antioxidant activity showed a significant variation (P < 0.001) among clones, whereas the interaction effect and nitrogen rate were non-significant at P < 0.05. Camellia sinensis exhibited antioxidant activity with an IC50 value of 70.25 ± 2.85 g/mL [41]. Clones in the study have a high competitive antioxidant inhibition percentage ranging between 51.9 and 66.5%. The highest antioxidant 66.5%, was obtained by clone 11/56, followed by clones 12/38 (62.7), 11/4 (59.2), BB-35 (56.07), and 6/8 (55.17%). The free radical scavenging IC50 value obtained in this study ranged from 0.311- 0.52 mg g^−1^. The lower IC50 value reflected the higher antioxidant activity of the test sample [42]. The high antioxidant effect of polyphenols is due to the presence of phenolic hydroxyl groups in their structures that make them protect free radical scavengers [43].

### 3.5 Sensory and quality assessment of green tea

The sensory evaluation of green tea was significantly affected by increasing nitrogen application rates (P < 0.01). The cup quality of green tea ranged from 19.23 to 29.88%. Clone 6/8 scored the most excellent cup quality at 0, 150, and 300 kg N ha^−1^, and the balance (i.e., sweet and bitter) of tasted clones ranked from the highest to the lowest in the following order (clones 6/8 > 11/4 > BB-35 > 11/56 > 12/38).

The interaction effect of liquor color was significantly different at P < 0.032, and the liquor color ranged from 18.78 to 23.95%. Clones 11/4 and 11/56 scored the highest (23.95 and 23.38%) green liquor color at 300 kg N ha^-1^, whereas clones 6/8 and BB-35 recorded the lightest (18.85 and 18.78%) green liquor color at 150 kg N ha^-1^ and control plots, respectively. The greenness of liquor is highly dependent on the amount of chlorophyll present in the leaf. Chlorophyll content gives the sense of a grassy taste in green tea products [44, 45]. The mean chlorophyll content of clones ranged from 11.09 to 32.63%, and clone 11/4 scored the highest (32.63%) in Southwest Ethiopia [32]. The dry-made tea appearance of green tea samples showed significant variation among clones at (P < 0.0001). Clone 11/4 scored the highest dry leaf appearance of 9.48% at 300 kg N ha^-1^, while clones BB-35, 6/8, and 12/38 had the lowest dry leaf appearance of 6.73, 7, and 7.35% at control plots, respectively. The infusion of green tea samples showed significant variation among clones (P < 0.02) and ranged from 13.15 to 17.08%. Clone 11/4 scored the highest (17.08%) green infusion, followed by clones 12/38 (16.7), 11/56 (16.65), 6/8 (16.45), and BB-35 (15.75). The aroma of green tea samples showed significant variation among clones (P < 0.0001). The amount of green tea aroma varies between 7.1 and 9.98%. Clone 6/8 has the highest (9.98%) green tea aroma, whereas clone 12/38 had the lowest (6.83, 6.95, and 7.1%) aroma at 300, 150 kg N ha^-1^, and control plots, respectively.

The overall quality of the tested clones showed significant differences at (P < 0.01). Clones 6/8 and 11/4 scored the highest overall quality above the total mean value (77.68%), whereas clones BB-35, 11/56, and 12/38 scored the lowest overall quality percentage below the mean value (Table 4). The overall quality of clones improved greatly when nitrogen levels were increased. Hence, the appropriate application of nitrogen fertilizer could balance the lipid metabolism and the formation of flavor/aroma origin compounds, which help to improve tea quality [46].

**Table 4:** Overall quality percentage of green tea.

Type Of Clone	Cup Quality 30%	Liquor 30%	Infusion 20%	Dry App. 10%	Aroma 10%	Overall Quality	Rank
6/8	29.43^a^	20.08^c^	15.48^b^	7.99^b^	9.98^a^	82.96^a^	1
11/4	24.48^b^	23.52^a^	16.65^a^	8.9^a^	8.61^b^	82.15^a^	2
11/56	21.46^c^	22.95^ab^	16.25^ab^	8.55^ab^	7.74^c^	76.95^b^	3
BB-35	23.48^b^	19.86^c^	14.38^c^	7.39^c^	8.3^b^	73.44^c^	4
12/38	19.98^d^	21.94^b^	15.82^b^	8.2^b^	6.96^d^	72.87^c^	5
Mean	23.77	21.67	15.72	8.21	8.32	77.68	

Different letters in the same columns indicate the difference between two means is Statistically significant (P < 0.01) for each of the treatment combinations.

The organoleptic assessment result of clones showed that clone 6/8 produced an excellent aroma, balanced bitterness, and a slightly sweeter cup than other clones in all nitrogen treatments. Clone 6/8 also contained a high level of 26.2 % polyphenols and a small amount of caffeine (2.35%), which is less than other clones in all nitrogen treatments, and this situation makes clone 6/8 more suitable for the production of green tea. However, clones 6/8 and BB-35 have light green liquor and a poor dry-made tea appearance. Typically, clones 6/8, BB-35, and 12/38 develop a poor dry leaf appearance after processing. The second-best clone, 11/4, has the best aroma, green liquor color, and an excellent marketable dry leaf appearance. Moreover, clone 11/4 contains (23.87%) total polyphenols and less caffeine content (2.57 %), and these criteria make clone 11/4 the second-best clone next to clone 6/8 for the production of green tea. Clone BB-35 has a medium aroma and contains more caffeine than other clones, whereas clones 11/56 and 12/38 have a poor aroma, and the cup has a bitter taste compared to other clones.

The cup quality correlates with aroma (r = 0.81), and the variable nitrogen rate is positive and weakly correlated with dry-made tea appearance and liquor color, but it has no significant correlation with other quality parameters (Table 5).

**Table 5:** Correlation among green tea quality parameter.

Parameter	Overall Quality	Type of Clone	Nitrogen rate	Cup Quality	Aroma	Dry leaf app.	Infusion	Liquor
Overall Quality	1							
Type of Clone	-0403∗∗	1						
Nitrogen rate	0.317∗	0.00∗∗∗	1					
Cup Quality	0.563∗∗∗	-0.705∗∗	0.10^ns^	1				
Aroma	0.569∗∗∗	-0.707∗∗∗	-0.07^ns^	0.81∗∗∗	1			
Dry Leaf app.	0.436∗∗∗	0.139^ns^	0.52∗∗∗	-0.127^ns^	-0.187 ^ns^	1		
Infusion	0.60∗∗∗	0.125^ns^	0.114 ^ns^	-0.15 ^ns^	0.033 ^ns^	0.267∗	1	
Liquor	0.425∗∗∗	0.296∗	0.285∗	-0.40∗∗	-0.347∗∗	0.606∗∗∗	0.533∗∗∗	1

∗, ∗∗, ∗∗∗ and ns indicate significance at the 0.05, 0.01, and 0.001 levels and non-significant.

The correlation coefficient between biochemical constitutes of green tea and nitrogen application was positively correlated with polyphenol, caffeine content, and leaf nitrogen content at (r = 0.387, r = 0.579, and r = 0.798), respectively (Table 6).

**Table 6:** Correlation among biochemistry constitution parameter.

Parameter	Nitrogen rate	Poly phenol	Anti oxidant	Leaf Nitrogen	Caffeine
Nitrogen rate	1				
Polyphenol	0.387∗∗∗	1			
Antioxidant	0.18^ns^	-0.255∗	1		
Leaf Nitrogen	0.798∗∗∗	0.58∗∗∗	-0.092 ^ns^	1	
Caffeine	0.579∗∗∗	-0.007 ^ns^	0.015 ^ns^	0.23∗	1

∗, ∗∗, ∗∗∗ and ns indicate significance at the 0.05, 0.01, and 0.001 levels, and non-significant.

## 4 Conclusion

Biochemical and organoleptic assessment results indicate that clone 6/8 has an excellent full aroma, balanced bitterness, and a slightly sweeter cup than other clones. These characteristics makes clone 6/8 more preferable to producing green tea. The second clone, 11/4, has the best aroma, marketable green liquor, and dry-made tea appearance. But, clone 11/4 is slightly bitterer than clone 6/8. Generally, the tested clones have a high antioxidant inhibition capacity. Furthermore, clone 6/8 contains high polyphenols and a small amount of caffeine in high nitrogen treatment, which is safe to feed small children with the daily-required amount of antioxidants and can be used to produce a more acceptable and high-quality green tea yield at 300 kg Nha^−1^.

## Declarations

### Author contribution statement

Adugna Debela, Yetenayet Bekele, Sultan Suleman: Conceived and designed the experiments; Performed the experiments; Analyzed and interpreted the data; Contributed reagents, materials, analysis tools or data.

Tesfaye Benti: Conceived and designed the experiments; Performed the experiments; Analyzed and interpreted the data; Contributed reagents, materials, analysis tools or data; Wrote the paper.

### Funding statement

This research did not receive any specific grant from funding agencies in the public, commercial, or not-for-profit sectors.

### Data availability statement

Data included in article/supp. material/referenced in article.

### Declaration of interest's statement

The authors declare no conflict of interest.

### Additional information

No additional information is available for this paper.
